# Perceived stress and psychological disorders in healthcare professionals: a multiple chain mediating model of effort-reward imbalance and resilience

**DOI:** 10.3389/fpubh.2023.1320411

**Published:** 2023-12-14

**Authors:** Yuanling Tao, Zhen Cheng, Chenxi Wang, Ting Liu, Mi Yan, Xiaohong Huang, Shasha Jian, Li Sun, Zongtao Chen

**Affiliations:** ^1^Health Management Centre, First Affiliated Hospital of Army Medical University, Chongqing, China; ^2^School of Nursing, Southern Medical University, Guangzhou, China

**Keywords:** perceived stress, anxiety, depression, effort-reward imbalance, resilience, serial multiple mediation, healthcare professionals

## Abstract

**Background:**

Healthcare professionals have shown more psychological disorders such as anxiety and depression due to the nature of work, which can cause job burnout, decrease the quality of medical services, and even endanger medical safety. The aim of the study is to explore the serial multiple mediating role of effort- reward imbalance and resilience between perceived stress and psychological disorders among healthcare professionals.

**Methods:**

A cross-sectional study was conducted in China from February to April 2023. A total of 2098 healthcare professionals at a tertiary general hospital was investigated by the following self-reported questionnaires: Hospital Anxiety and Depression Scale (HADS), Perceived Stress Scale (PSS), The Effort-Reward Imbalance (ERI), Healthcare professionals Resilience Scale (MSRS).

**Results:**

Anxiety and depression are interrelated (*r* = 0.362, *p* < 0.01), and they were positively related to perceived stress (*r* = 0.640/0.607, *p* < 0.01) and ERI (*r* = 0.422/0.383, *p* < 0.01), and negatively related to resilience (*r* = −0.343/−0.320, *p* < 0.01). After controlling demographic factors, the variance in anxiety and depression was explained by perceived stress was 37.7 and 35.0%. Bootstrap analyses examining the pathway of perceived stress-ERI-resilience-anxiety revealed significant direct effects [*B* = 0.560, 95%CI (0.528, 0.591)], as well as indirect effects mediated independently by ERI [*B* = 0.045, 95%CI (0.029, 0.060)], resilience [*B* = 0.031, 95%CI (0.017, 0.047)], or a combination of both [B = 0.004, 95%CI (0.002, 0.007)]. Similarly, in the path of perceived stress-ERI-resilience-anxiety-depression, significant direct effects were found [*B* = -0.310, 95%CI(0.265, 0.351)], along with indirect effects mediated individually by ERI [*B* = 0.033, 95%CI(0.013, 0.052)], resilience [*B* = 0.014, 95%CI (0.001, 0.028)], and anxiety [*B* = 0.218, 95%CI (0.190, 0.246)], or by both or three together (*B* = 0.032).

**Conclusion:**

This study proved the hypothesis that ERI and resilience played a mediating role in perceived stress and psychological disorders, revealed the potential mechanism of anxiety in stress and depression, and proposed a solution for perceived stress to psychological distress, which can provide a basis for the intervention of healthcare professionals in the face of mental health crisis.

## 1 Introduction

Healthcare professionals are in short supply, with high work intensity, heavy workload and high risk, requiring them to master the operation in various working environments, thus they are facing great pressure and job burnout (1, 2). Especially after the normalization of the COVID-19 epidemic, higher requirements are put forward for their physical and psychological quality. In the case of environmental changes and self-adjustment imbalances, they would have a lot of psychological disorders, such as anxiety and depression. A recent survey of healthcare professionals indicated, the incidence of anxiety and depression was 15–20% (3). A survey of Tunisian residents found that 43.6% of participants had definite anxiety and 30.5% had definite depression (4). A cross-sectional investigation of 1,679 healthcare workers at 27 hospitals in Heilongjiang Province found that their anxiety and depression levels were higher than those of the general Chinese population (5). According to a study of psychiatrists in China, the prevalence of depression was 17.74% (6). Notably, during the COVID-19 pandemic, Medical and nursing staff in Wuhan had mental health disturbances below the threshold in 36.9% of cases (7). Anxiety and depression of the healthcare professionals may further lead to poor quality of life, and even suicide. Meanwhile, the work ability is greatly reduced, the job burnout is increased, the quality of medical service is lowered, and even the medical security is threatened (3, 5). Therefore, after experiencing the fight against the COVID-19 epidemic, the current mental state of healthcare professionals and its occurrence mechanism under the normalization of the epidemic deserve more attention.

Anxiety and depression are common psychological disorders that are highly related and often comorbid (8, 9). Anxiety was proven to be a predictor of depression and preceded the onset of depression (10). Meanwhile, previous studies have demonstrated that the anxiety and depression of healthcare professionals were affected by perceived stress and resilience (11). Perceived stress is a factor that causes anxiety and depression, and higher levels of perceived stress have a negative impact on mental health (12). Many studies have documented the protective effect of resilience on mental health (13, 14). Resilience can be effectively adjusted when individuals face setbacks or adversities, and has a positive impact on relieving anxiety and depression (15). The Effort-Reward Imbalance (ERI) is one of the specific factors that affect the link between job stress and psychological health, and those who experience the high effort and low reward, as well as those with high over-commitment, have a much higher threat of depression at follow-up (16).

Perceived stress is how much a person considers certain situations to be stressful, and it may help to explain the connection between job stress and psychological symptoms (17). High perceived stress does lead to strong negative emotions among healthcare professionals (18). The high perceived stress of healthcare professionals comes not only from life stressors, but also from occupational stressors, such as the effort-reward imbalance in job Surrounding (19). Studies have shown that resilience could mitigate the effects of perceived stress on depression through direct and indirect pathways during the COVID-19 pandemic (20).

The Effort-Reward Imbalance (ERI) model, which was introduced by German physiologist Siegrist (21), is a classic theory of job stress and has been widely utilized in occupational mental health. The model states that when there exists an imbalance between the input of time and energy into work and the output of money, esteem, cognition, and job opportunities from the job, that is high effort and low reward, it can result in detrimental emotions. These emotions, in turn, influence the ongoing response of the vegetative nervous system, giving rise to insomnia and potentially psychological issues (22). Many studies have confirmed that employees with high effort and low reward report more mental disturbance (23, 24). ERI was highly correlated with psychological states (16, 25, 26). In addition, a study found that ERI was positively related to perceived stress (27). Studies have shown the relationship between ERI and resilience. In addition, resilience can weaken the connection between ERI and depression, which is a potential protective factor against psychological distress (28).

Resilience is a personality trait that aids individuals in handling with adversity and making good adjustment and development (29). Many studies have confirmed that resilience as a protective impact on mental health (14, 30). Therefore, some scholars believed that the cultivation of resilience occurs within the dynamic interplay of adverse life events and protective factors. Individuals can adjust their resilience to diminish the impact, and to maintain their positive mental traits when faced with negative life events, including effort- reward imbalance (31). At the same time, resilience significantly regulated the impact of perceived stress on depression (15).

To examine the mediating mechanism of perceived stress on psychological disorders based on occupational factors and personal characteristics, and propose intervention measures, is the focus of this study. Previous studies have demonstrated the relationship between perceived stress, ERI, resilience, anxiety and depression (12, 13, 15, 16, 27, 28). Although some studies have confirmed the mediation role of resilience in work stress and mental health (28, 32–34), no studies have explored whether effort-reward imbalance and resilience modulate perceived stress to influence the occurrence of anxiety and depression. In addition to the co-occurrence of anxiety and depression, dozens of studies have revealed that anxiety symptoms frequently precede and predict the onset of depressive symptoms (35, 36). However, the intermediation of anxiety between perceived stress and depression is unclear. The relationships between perceived stress, ERI, resilience, anxiety and depression were thus examined through a serial multiple mediation model. We hypothesize that (I) perceived stress, ERI, resilience, anxiety and depression would be interconnected; (II) the mediation of ERI and resilience would affect the relationship between perceived stress and depression/anxiety; (III) anxiety could act as a mediator between perceived stress and depression.

## 2 Methods

### 2.1 Participants and procedures

A cross-sectional study based on self-reported questionnaires was conducted between February and April 2023, which was just after the opened up to the COVID-19 epidemic in China. By convenience sampling, a total of 2098 staff members in a tertiary general hospital were selected to fill out an online questionnaire. The inclusion criteria: (1) The age range encompasses those aged 18 to 65; (2) working in the hospital; (3) work for more than one year; (4) Voluntary participation in the investigation. The exclusion criteria is support personnel, who are responsible for transport, rear services and other work. In this study, the investigators conducted unified training, and participants gave informed consent to fill in the form. To make sure the filling is complete, it is necessary to answer all questions before submitting the online questionnaire. This study was given approval by the Ethics Committee of the First Affiliated Hospital of Army Medical University ((B)KY2023088).

### 2.2 Measurements

#### 2.2.1 Demographic data

Basic demographic information consists of gender (male or female), age (years), department (Front-line epidemic department or others), educational level (junior college to doctor), personnel category (medical and nursing staff, or others), years of working (less than 3 years, 3–5 years, 5–10 years, more than 10 years), technical title (primary, intermediate, deputy senior, or senior), whether they have been infected with COVID-19, working hours during the pandemic (less than 8 h, 8–10 hours, 11–12 h, over 12 h), and the number of participating in major anti-epidemic missions (zero, one time, one to three times, and over three times).

#### 2.2.2 Hospital anxiety and depression scale (HADS)

The Scale was created in 1983 and put together by Zigmond and Snaith (37). It is a screening tool used to measure symptoms of anxiety and depression. Participants were informed that the inquiries were centered around their mental state during the past 2 weeks. There are 14 items on the scale, with 7 of them being for depression and 7 for anxiety. A four-point Likert scale (0–3) is used to rate all items, and higher scores show more severe anxiety or depression. Anxiety and depression are considered if any of the subscales are below 8 points. The anxiety and depression subscale had a reliability of 0.83 and 0.81 for internal consistency (37, 38).

#### 2.2.3 Perceived stress scale (PSS)

It was produced by Dr. Cohen, an American psychologist, in 1983 and translated it to Chinese by Yang Tingzhong in 2003 (39). This is an internationally accepted and widely used measurement tool, which is utilized to assess the degree of a person’s perception of life stress. The study adopted the 14-item version, including two dimensions of tension and feeling out of control, with five options for each item: never, almost never, sometimes, often, and always, corresponding to a score of 0–4. The total score ranges from 0 to 56 points, and the higher the score, the more pressure there is. The internal consistency reliability of the scale was 0.954 (40).

#### 2.2.4 The effort-reward imbalance (ERI)

This scale was compiled by German sociologist Siegrist in 1996 and introduced into China by Li Jian in 2004, to evaluate workplace stress (41). There are 23 items in total, which are divided into three parts: effort (6 items), reward (11 items), and over-commitment (6 items). The “effort” score is 6 to 30 points, the “reward” score is 11 to 55 points, and the “over-commitment” score is 6 to 24 points. ERI is the ratio between the total effort score (E) and the total reward score (R) divided by c, where C is the ratio of the number of effort dimension items to the number of reward items, i.e., ERI = E/(R × c). If ERI>1, it can be identified as high effort and low reward, and ERI ≤1 is regarded as low effort and high reward. Those whose score in the top third of the over-commitment dimension are considered to be overcommitted. The reliability of effort, reward and overcommitment ranged between 0.7 to 0.8 (42).

#### 2.2.5 Healthcare professionals resilience scale (HPRS)

This questionnaire was proposed by Zhu Houqiang in 2016 to measure the level of resilience among medical workers in China (43). It includes 18 items in 4 dimensions: decision coping, interpersonal relationship, rational thinking and flexible adaptation, and the number of items in the four dimensions are 6,4,4,4, respectively. Each entry was scored on a 5-point Likert scale, ranging from completely disagree to completely agree on a scale of 1 to 5. The higher the total score is, the greater the level of resilience. With a Cronbach’s α coefficient of 0.907, this scale was reliable and valid (43).

### 2.3 Statistical analysis

Data analysis was carried out using IBM SPSS 24.0 and Amos 26.0 statistical software. Demographic characteristics were analyzed through descriptive analysis, and each scale scores were portrayed in the form of means and standard deviations. The percentage (%) was used to express the count of data. Pearson correlation analysis was utilized to examine the relation of perceived stress, ERI, resilience, anxiety and depression. To assess the relationship between perceived stress, ERI, resilience, anxiety, and depression, a multiple linear regression model was constructed after controlling for socioeconomic factors.

The mediation of ERI, resilience and anxiety were examined using the structural equation model. The maximum likelihood method was used to estimate the parameters of the covariance matrix. The model’s suitability of the data was determined by selecting indicators such as *χ*^2^/df, SRMR, GFI, CFI, TLI, AGFI, NFI and RMSEA. Through bootstrapping analysis of 5,000 samples, 95% confidence interval (CI) was obtained to determine the direct and indirect effects. The significance of total effects, direct effects, and indirect effects depends on whether zero is existed in the 95% CI.

## 3 Results

### 3.1 Common method bias

The methods of anonymous assessment, reasonable setting of question order and length, and reverse grading of some items were used to control the quality of questionnaire collection, so as to decrease the methodological bias caused by self-reported surveys. To examine the deviation of common methods, the Harman single factor method was employed. The findings showed that there were 10 common factors with eigenvalues above 1, which explained 65.33% of the variation. The explained percentage of variance of the first common factor was 27.71%, which fell short of 40%, so it can be considered that there was no significant common method bias in this study.

### 3.2 Characteristics of participants

A total of 2,098 healthcare professionals was surveyed availably in this study, including 439 males (20.9%) and 1,659 females (79.1%). Detailed information is shown in Table 1.

**Table 1:** The demographic characteristics of healthcare professionals (*n* = 2098).

Characteristics	*N* (%)	Characteristics	*N* (%)
Gender		Professional title	
Male	39 (20.9)	Primary	1036 (49.4)
Female	1,659 (79.1)	Secondary	879 (41.9)
Age		Deputy senior	170 (8.1)
<35 years old	1,288 (61.4)	Senior	13 (0.6)
36–50 years old	767 (36.6)	Affected by COVID-19	
>50 years old	43 (2.0)	No	233 (11.1)
Department		Yes	1865 (88.9)
COVID-19 frontline department	229 (10.9)	Working hours during the COVID-19 pandemic	
Others	1869 (89.1)		
Education		<8 h	601 (28.6)
Junior college and below	138 (6.6)	8–10 h	926 (44.1)
Undergraduate	1,472 (70.2)		
Graduate	488 (23.3)	11–12 h	110 (5.2)
Job category		>12 h	461 (22.0)
Doctors and nurses	1,351 (64.4)	Frequency of anti-epidemic missions	
Others	747 (32.6)	Zero	1,636 (78.0)
Length of work		One	334 (15.9)
<3 years	315 (15.0)	One to three	99 (4.7)
3–5 years	274 (13.1)	More than three	29 (1.4)
5–10 years	528 (25.2)		
>10 years	981 (46.8)		

### 3.3 The prevalence and correlation of various variable

It was found that anxiety had a positive correlation with depression (r = 0.362). Perceived pressure was associated positively with anxiety and depression (r = 0.640 and 0.607). Anxiety, depression, perceived pressure, and ERI were negatively related to resilience, respectively. Anxiety, depression, and perceived pressure were positively related to ERI, respectively. The above results are shown in Table 2.

**Table 2:** Description of variables and correlation analysis results.

Variables	Mean	SD	Depression	Anxiety	Perceived pressure	Resilience	ERI
1.Depression	4.46	3.28	1				
2.Anxiety	4.88	3.44	0.362**	1			
3.Perceived pressure	22.15	7.67	0.607**	0.640**	1		
4.Resilience	75.70	12.39	−0.320**	−0.343**	−0.403**	1	
5.ERI	0.72	0.44	0.383**	0.422**	0.451**	−0.343**	1

^**^P<0.01.

### 3.4 Multiple linear regression of anxiety and depression

With anxiety and depression as dependent variables respectively, hierarchical logistic regression was employed. The collinear analysis showed that the tolerance value was between 0.51 and 0.96, and the VIF value was about 1.00 ~ 2.00. It showed that the independent variables have no serious collinear problem.

In the linear regression of anxiety: after adjusting demographic factors like age and gender, four models were established by adding the variables of perceived pressure, resilience, and ERI gradually, as illustrated in Table 3. The results showed that perceived pressure explained 37.7% of the variance in anxiety, and perceived pressure, resilience, and ERI all were significant regression factors for anxiety (*β* = 0.538, −0.067 and 0.147, *p* < 0.001).

**Table 3:** Multiple linear regression of anxiety in healthcare professionals.

Independent variable	Model 1			Model 2			Model 3			Model 4		
	*β*	*t*	*P*	*β*	*t*	*P*	*β*	*t*	*P*	*β*	*t*	*P*
Gender	0.068	3.008	0.003	0.010	0.543	0.587	0.013	0.735	0.463	0.019	1.108	0.268
Age	−0.089	−3.201	0.001	−0.008	−0.375	0.708	−0.002	−0.071	0.943	−0.003	−0.141	0.888
Department	0.016	0.707	0.480	0.015	0.849	0.396	0.014	0.813	0.417	0.012	0.677	0.499
Education	0.033	1.306	0.192	0.026	1.307	0.191	0.023	1.176	0.240	0.012	0.641	0.522
Job category	0.097	4.249	<0.001	0.049	2.725	0.006	0.041	2.318	0.021	0.032	1.830	0.067
Length of work	0.136	4.683	<0.001	0.018	0.810	0.418	0.020	0.890	0.374	0.011	0.512	0.609
Professional title	−0.031	−1.127	0.260	0.016	0.720	0.472	0.011	0.503	0.615	0.006	0.294	0.768
Affected by COVID-19	0.047	2.171	0.030	0.025	1.449	0.147	0.021	1.265	0.206	0.016	0.970	0.332
Working hours during the COVID-19 pandemic	0.037	1.638	0.102	0.021	1.216	0.224	0.020	1.130	0.258	0.008	0.436	0.663
Frequency of anti-epidemic missions	0.030	1.365	0.172	0.011	0.608	0.543	0.007	0.421	0.674	0.008	0.487	0.626
Perceived pressure				0.631	36.649	<0.001	0.594	31.918	<0.001	0.538	27.339	<0.001
Resilience							−0.094	−5.086	<0.001	−0.067	−3.652	<0.001
ERI										0.147	7.692	<0.001
*F*	8.510			134.816			127.210			125.251		
*p*	<0.001			<0.001			<0.001			<0.001		
Adjusted *R^2^*	0.035			0.412			0.419			0.435		
R^2^-changes	0.035			0.377			0.007			0.016		

“ERI” means “the effort-reward imbalance.”

In the linear regression of depression: after controlling demographic factors, the variables of perceived pressure, resilience, ERI, and anxiety were added step by step to build five models, as displayed in Table 4. The results showed that perceived pressure explained 35.0% of the variance in depression, and perceived pressure, resilience, ERI, and anxiety all had a regressive effect on depression (*β* = 0.316, −0.043, 0.057 and 0.395, *p* < 0.05).

**Table 4:** Multiple linear regression of depression in healthcare professionals.

Independent variable	Model 1			Model 2			Model 3			Model 4			Model 5		
	*β*	*t*	*P*	*β*	*t*	*P*	*β*	*t*	*P*	*β*	*t*	*P*	*β*	*t*	*P*
Gender	−0.027	−1.190	0.234	−0.083	−4.570	<0.001	−0.008	−4.414	<0.001	−0.075	−4.168	<0.001	−0.082	−4.964	<0.001
Age	−0.063	−2.254	0.024	0.015	0.667	0.505	0.021	0.954	0.340	0.020	0.909	0.363	0.021	1.042	0.298
Department	0.044	1.945	0.052	0.043	2.379	0.017	0.042	2.352	0.019	0.040	2.259	0.024	0.036	2.164	0.031
Education	0.008	0.377	0.737	0.002	0.077	0.938	−0.001	−0.051	0.959	−0.009	−0.467	0.641	−0.014	−0.769	0.442
Job category	0.064	2.768	0.006	0.017	0.917	0.359	0.010	0.532	0.595	0.003	0.145	0.885	−0.010	−0.598	0.550
Length of work	0.178	6.139	<0.001	0.065	2.793	0.005	0.067	2.877	0.004	0.060	2.601	0.009	0.056	2.602	0.009
Professional title	−0.089	−3.169	0.002	−0.043	−1.931	0.054	−0.048	−2.145	0.032	−0.051	−2.324	0.020	−0.054	−2.635	0.008
Affected by COVID-19	0.052	2.359	0.018	0.030	1.701	0.089	0.027	1.530	0.126	0.023	1.304	0.192	0.016	1.010	0.312
Working hours during the COVID-19 pandemic	0.049	2.177	0.030	0.034	1.894	0.058	0.033	1.817	0.069	0.023	1.289	0.197	0.020	1.215	0.225
Frequency of anti-epidemic missions	0.004	0.176	0.860	−0.015	−0.849	0.396	−0.018	−1.030	0.303	−0.018	−0.922	0.321	−0.021	−1.274	0.203
Perceived pressure				0.607	34.322	<0.001	0.571	29.873	<0.001	0.528	25.922	<0.001	0.316	14.372	<0.001
Resilience							−0.090	−4.745	<0.001	−0.069	−3.628	<0.001	−0.043	−2.409	0.016
ERI										0.115	5.824	<0.001	0.057	3.080	0.002
Anxiety													0.395	18.845	<0.001
*F*	7.422			117.645			110.830			106.528			141.094		
*p*	<0.001			<0.001			<0.001			<0.001			<0.001		
Adjusted *R^2^*	0.030			0.380			0.389			0.395			0.483		
*R*^2^-changes	0.030			0.350			0.009			0.006			0.088		

“ERI” means “the effort-reward imbalance.”

### 3.5 Structural equation model for mediation effect: developing and evaluating

**Figure 1:**
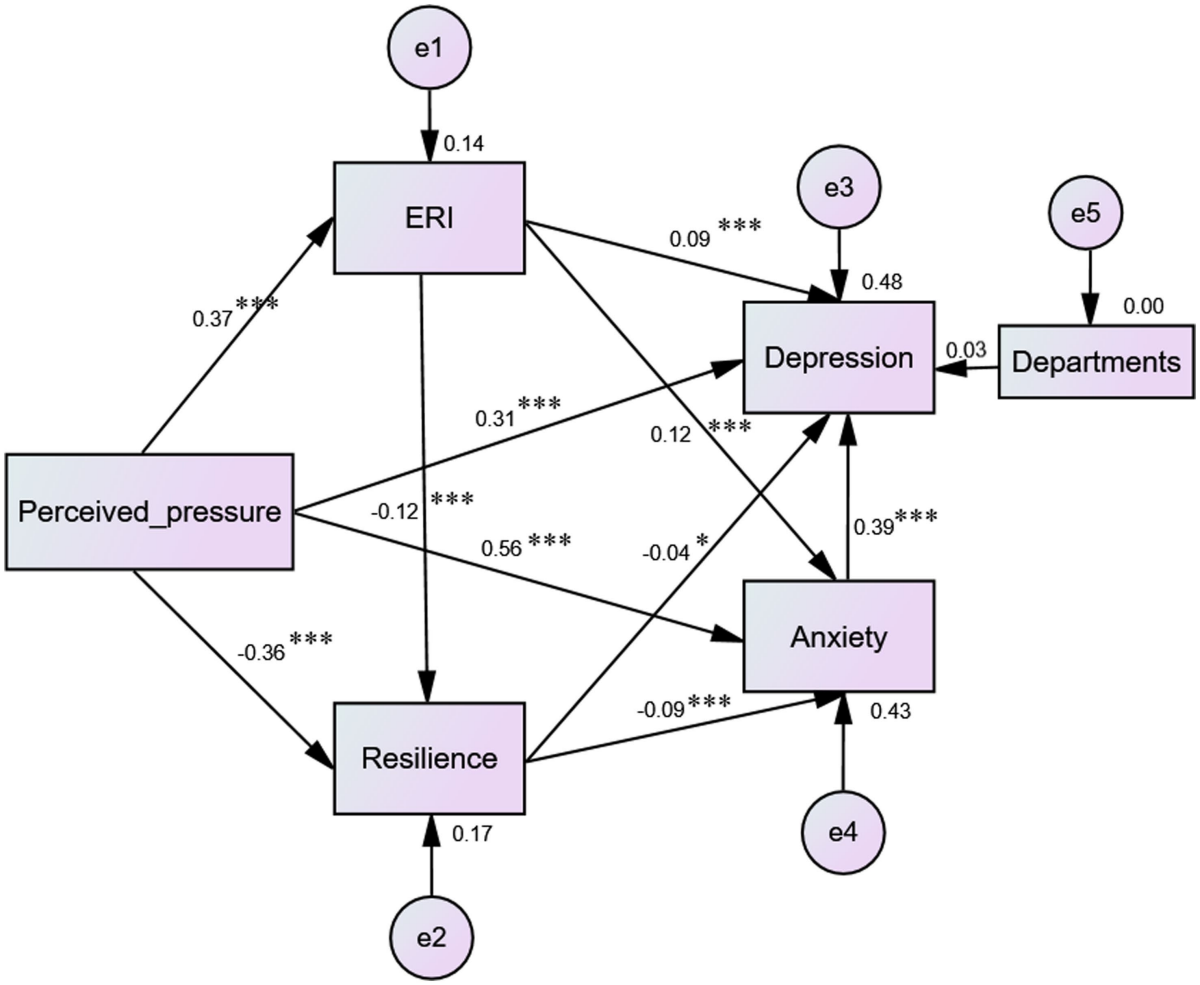
Chain mediation model of perceived pressure, resilience, ERI, anxiety and depression.

In this model, gender, department, Length of work, professional title and other control variables were added, and the optimal model was obtained after the screening and comparison, as shown in Figure 1. The fitness indicators of structural equation model: *χ*^2^/df = 1.762, SMRM = 0.018, GFI = 0.999, CFI = 0.999, TLI = 0.996, AGFI = 0.994, NFI = 0.998 and RMSEA = 0.019.

The deviation-corrected percentile Bootstrap method with 5,000 iterations was utilized to examine the mediation effect. The findings revealed that the 95% CI of the total, direct and indirect effects of perceived pressure and anxiety/depression did not encompass 0, which indicates a significant mediating influence (*p* < 0.05). Moreover, the effect of chain mediation was also significant (*p* < 0.05). On that path of perceived pressure to anxiety, ERI and resilience played a small mediating role (mediation effect size is 0.045/0.031), and the sequential mediation effect was significant (mediation effect size is 0.004). The total indirect effect accounted for 12.5% of the total effect. On that path of perceived pressure to depression, ERI, resilience and anxiety presented partial mediating effects (mediation effect size is 0.033/0.014/0.218), and sequential mediation effect demonstrated significant (mediation effect size is 0.002/0.017/0.012/ 0.001). The total indirect effect accounted for 48.9% of the total effect. Table 5 provided detailed information.

**Table 5:** Bootstrap analysis of the significance test of the mediation effect.

Path		Effect size	SE	Bias-corrected 95%CI		*P*
				Lower	Upper	
Perceived pressure → Anxiety	Total effects	0.640	0.012	0.617	0.662	<0.001
	Direct effects	0.560	0.016	0.528	0.591	<0.001
	Ind1: Perceived pressure →ERI → Anxiety	0.045	0.008	0.029	0.060	0.001
	Ind2: Perceived pressure → Resilience → Anxiety	0.031	0.008	0.017	0.047	<0.001
	Ind3: Perceived pressure →ERI → Resilience → Anxiety	0.004	0.001	0.002	0.007	<0.001
	Total indirect effect	0.080	0.011	0.060	0.101	<0.001
Perceived pressure → Depression	Total effects	0.607	0.014	0.579	0.633	<0.001
	Direct effects	0.310	0.022	0.265	0.351	<0.001
	Ind1: Perceived pressure → ERI → Depression	0.033	0.010	0.013	0.052	<0.001
	Ind2: Perceived pressure →Resilience →Depression	0.014	0.007	0.001	0.028	0.038
	Ind3: Perceived pressure → Anxiety →Depression	0.218	0.014	0.190	0.246	<0.001
	Ind4: Perceived pressure → ERI → Resilience →Depression	0.002	0.001	0.000	0.005	0.025
	Ind5: Perceived pressure → ERI → Anxiety →Depression	0.017	0.003	0.011	0.024	0.001
	Ind6: Perceived pressure → Resilience → Anxiety →Depression	0.012	0.003	0.007	0.019	<0.001
	Ind7:Perceived pressure → ERI → Resilience → Anxiety →Depression	0.001	0.001	0.001	0.003	<0.001
	Total indirect effect	0.297	0.016	0.266	0.331	<0.001

## 4 Discussion

This study confirmed the relationship between perceived stress, ERI, resilience and anxiety/depression. The five factors were proved to be connected to each other, and perceived stress, ERI, and resilience were all predictors of anxiety and depression. In the multi-chain mediating model, depression was affected by the departments. As the departments in this study were divided into COVID-19 frontline department and others, it is well known that, different from ordinary departments, frontline health care workers show a high level of stress and emotional symptoms (44). After adjusting control variables, the model and data were well aligned, and the results confirmed all the hypotheses in this study. The impact of perceived stress on psychological distress was both direct and indirect. In addition, perceived stress and anxiety/depression were mediated in part by ERI and resilience, and anxiety was the primary mediator between perceived stress and depression. For the first time, occupational factors were included in this study to examine the role of ERI in the mediation of perceived stress and anxiety/depression, and the chain mediating role of ERI with resilience. Meanwhile, the potential mediating effect of anxiety to depression was also discussed. Exploring the multiple linkage mechanism of self-perception stress, workplace environment and individual trait on psychology is significant to prevent and treat the psychological distress of healthcare professionals.

In this study, perceived stress had a positive correlation with anxiety and depression (*r* = 0.640/0.607), explained more than a third of the difference in anxiety/depression (37.7%/35.0%), and had a significant impact on both anxiety and depression through direct and indirect means (direct effects: *B* = 0.560/0.310; indirect effects: *B* = 0.080/0.297; *p* < 0.001), which was in line with previous researches (32, 34, 45, 46). The observed association between perceived stress and anxiety/depression provided support for the influence of life and work-related stressors on the mental well-being of healthcare professionals. The perception of stress in individuals is influenced by various factors, such as the nature of the event, behavioral and genetic factors, past experiences, and personal resources. When individuals perceive high levels of stress that surpass their coping abilities, it can elicit a variety of emotional responses (47). Because stress can cause changes in serotonin, which can disrupt mood regulation, this is also a key pathogenesis of depression (48). Meanwhile, stress has the potential to inhibit the immune system (49), leading to alterations in immune cytokine levels that can result in adverse emotions (50). Health workers have heavy tasks, immense responsibilities and burdensome workloads, often undergoing strict and prolonged training to attain their career goals, especially during the COVID-19 pandemic, the alterations in work and family routines, the potential risk of infection, the dynamic adjustment of roles, etc., make them suffer more negative emotions from professional factors. Therefore, enhancing the mental well-being of healthcare professionals is essential to find solutions from the internal source, such as reducing perceived stress and analyzing how it directly and indirectly affect anxiety and depression through occupational factors and personal resources.

According to the chain mediation model, ERI and resilience acted as mediators between perceived stress and anxiety/depression, either alone or in combination. In the perceived stress to anxiety pathway, the indirect impact of ERI and resilience is 12.5% of the total impact. In the perceived stress to depression pathway, the indirect effects of ERI, resilience and anxiety accounted for 48.9% of the total effect. There was a certain mediating role that ERI and resilience played. ERI refers to the imbalance between the effort and the reward of healthcare professionals in the work, that is, the effort is greater than the reward. It has a negative effect on mental state, as ERI amplifies the medical worker’s perception of stress, thus exacerbating psychological distress. China’s recent healthcare reform aims to effectively alleviate the medical burden. However, in practice, it has introduced new imbalances and significantly jeopardized the interests of healthcare professionals. They now find themselves working longer hours for the same compensation as before (51, 52). Furthermore, inadequate incentives within hospitals and strained doctor-patient relationships, including nurse–patient relationships, have substantially heightened the psychological burden on healthcare workers (53). Therefore, hospital managers should pay attention to the efforts and rewards of healthcare professionals, provide better organizational support, welfare and incentive measures to avoid the psychological burden caused by imbalance.

Resilience had a buffering effect on perceived stress and psychological distress, which was in accordance with previous researches (32, 34, 54). It not only regulated the relationship between perceived stress and anxiety/depression, but also adjusted the correlation between ERI and anxiety/depression. Simultaneously, resilience and ERI collaboratively serve as mediating factors that influence the impact of perceived stress on anxiety/depression. Resilience is an important psychological trait, and resilient people typically have more social support and more positive emotional regulation, thus mitigating the negative effects of perceived stress (55). When there is an imbalance between effort and reward at work, people with higher resilience will show a better buffer effect to grasp the balance (28). Effective psychosocial interventions can provide enduring enhancements in resilience, thereby offering healthcare professionals the opportunity to benefit from the essential protective factors for mental well-being (56). Therefore, specific strategies and interventions to build resilience may be particularly important, such as actively promoting self-care, social support and community engagement among healthcare professionals to develop key components of resilience.

Another point of interest in this study is that anxiety played more than half of the mediating effect of perceived stress on depression. Simultaneously, anxiety, ERI and resilience, respectively, or together formed chain mediation. As mentioned in the previous paragraph, perceived stress and ERI positively promote anxiety, and resilience buffers the influence of stress on anxiety, so it constituted a sequential three-factor chain intermediary. Numerous studies have established a strong association between anxiety and depression, with anxiety often serving as a precursor to depressive symptoms (57). Anxiety symptoms frequently preceded and predicted the emergence of depressive symptoms, as proven by dozens of studies (36). In line with a multiple pathway model, three distinct pathways connecting anxiety and depression have been identified. One of these pathways indicates that untreated anxiety disorders carry a higher risk for developing depression (58). Interpersonal relationships represent a potential mechanism that could establish a connection between anxiety and depression (36). Generalized anxiety can have adverse effects on interpersonal relationships, leading individuals to exhibit submissive behaviors that are a manifestation of passivity and powerlessness. This decline in interpersonal status has been suggested to be associated with the onset of depression (59). Therefore, monitoring and intervening for anxiety may help reduce depression.

Although the current study presents fresh insights into the mechanisms of perceived stress to psychological distress, there are some limitations that should be noted: firstly, all the healthcare professionals were from one general hospital, so it is prudent to generalize the results to other hospitals in China; secondly, whether this study is applicable to other populations affected by the epidemic deserves further investigation and exploration (60); thirdly, the psychological indicators are dynamic, and the cross-sectional design made it difficult to establish causal attributions between perceived stress, anxiety and depression. Future studies are still needed to track these factors longitudinally and control for other mediating factors specifically, while including other positive indicators; finally, this study simply explored the mediating effects of anxiety and depression, but some studies showed that there was a bidirectional path between anxiety and depression, rather than a single directivity (61). Future studies should further explore the potential circular mechanism between the anxiety and depression.

## 5 Conclusion

In sum, the current study supported the relationship among perceived stress, ERI, resilience, anxiety and depression, identified the role of ERI and resilience as mediators between perceived stress and psychological status, and revealed the potential mechanism of anxiety in stress and depression, which to further analyzed the causes of perceived stress leading to negative emotions. Previous studies have discussed the causes of anxiety and depression from a psychological perspective, but few have considered the solution of perceived stress to psychological distress from the perspective of occupational nature and positive personal traits. This study can provide evidence for the intervention of the healthcare professionals when encountered mental health crisis.

## Data availability statement

The raw data supporting the conclusions of this article will be made available by the authors, without undue reservation.

## Ethics statement

The studies involving humans were approved by the Ethics Committee of the First Affiliated Hospital of the Army Medical University. The studies were conducted in accordance with the local legislation and institutional requirements. The participants provided their written informed consent to participate in this study.

## Author contributions

YT: Formal analysis, Investigation, Methodology, Project administration, Writing – original draft. ZhC: Investigation, Writing – review & editing, Validation. CW: Writing – review & editing, Methodology. TL: Investigation, Writing – review & editing. MY: Investigation, Writing – review & editing. XH: Investigation, Writing – review & editing. SJ: Investigation, Writing – review & editing. LS: Project administration, Supervision, Writing – review & editing. ZoC: Project administration, Supervision, Writing – review & editing.
